# Association Between Weight Change and Leukocyte Telomere Length in U.S. Adults

**DOI:** 10.3389/fendo.2021.650988

**Published:** 2021-07-28

**Authors:** Yiling Zhang, Ziye Xu, Yiling Yang, Shanshan Cao, Sali Lyu, Weiwei Duan

**Affiliations:** ^1^Department of Bioinformatics, School of Biomedical Engineering and Informatics, Nanjing Medical University, Nanjing, China; ^2^Jiangsu Key Lab of Cancer Biomarkers, Prevention and Treatment, Collaborative Innovation Center for Personalized Cancer Medicine, Nanjing Medical University, Nanjing, China; ^3^Department of Biostatistics, Center for Global Health, School of Public Health, Nanjing Medical University, Nanjing, China

**Keywords:** aging, telomere length, weight change, obesity, NHANES

## Abstract

**Objective:**

To investigate the association of dynamic weight change in adulthood with leukocyte telomere length among U.S. adults.

**Methods:**

This study included 3,886 subjects aged 36-75 years from the National Health and Nutrition Examination Survey (NHANES) 1999-2002 cycle. Survey-weighted multivariable linear regression with adjustments for potential confounders was utilized.

**Results:**

3,386 individuals were finally included. People with stable obesity had a 0.130 kbp (95% CI: 0.061-0.198, *P*=1.97E-04) shorter leukocyte telomere length than those with stable normal weight (reference group) during the 10-year period, corresponding to approximately 8.7 years of aging. Weight gain from non-obesity to obesity shortened the leukocyte telomere length by 0.094 kbp (95% CI: 0.012-0.177, *P*=0.026), while normal weight to overweight or remaining overweight shortened the leukocyte telomere length by 0.074 kbp (95% CI: 0.014-0.134, *P*=0.016). The leukocyte telomere length has 0.003 kbp attrition on average for every 1 kg increase in weight from a mean age of 41 years to 51 years. Further stratified analysis showed that the associations generally varied across sex and race/ethnicity.

**Conclusions:**

This study found that weight changes during a 10-year period was associated with leukocyte telomere length and supports the theory that weight gain promotes aging across adulthood.

## Introduction

Telomeres are highly regulated complexes consisting of G-rich sequences and protective telomere-binding proteins, shorten with cell division in somatic cells (1, 2). It protects the end of chromosome against deterioration and fusion, playing a pivotal role in nuclear genome stabilization. Dysfunctional telomeres could elicit DNA damage checkpoint responses that trigger telomere-initiated senescence (3). The length of human telomeres generally shortens as people get older during adulthood (4, 5). For decades, a large number of experimental and observational studies have revealed that telomere length (TL) is associated with age-related diseases (6, 7), and even cancer risks (8–10). Inflammation, oxidative stress, hypoxia and unhealthy habits can cause DNA damage and telomerase dysfunction, leading to telomere attrition (11). TL in leukocytes has been well documented as a proxy for relative TL in other tissues due to the high correlations between them, and this parameter is easily measured in blood samples (2, 12). Therefore, leukocyte telomere length (LTL) could be considered as an underlying biomarker of age-related disorders.

Obesity has become an emerging epidemic over the last 50 years. In 2016, more than 1.9 billion adults were overweight (BMI ≥ 25 kg/m^2^) and of these, over 650 million were people with obesity (BMI ≥30 kg/m^2^), a number that has nearly tripled since 1975 (13). Obesity has been linked with increased risks of many chronic noninfectious diseases, such as type 2 diabetes (14), certain types of cancers (15, 16) and premature disability (17). High systemic inflammation and oxidative stress are observed in obesity (18). Thus, it has been proposed that obesity may accelerate telomere shortening. Previous epidemiologic studies did not yield completely consistent results regarding the association between obesity and LTL; that is, some confirmed this association (19, 20), while others did not (21–23). However, all of these studies measured body weight at a single time point, ignoring the changing trend in body weight over time. Weight gain across adulthood has been recognized as a risk factor for cardiovascular disease, diabetes, cancer and mortality (24–27). Thus, it is necessary to assess the long-term effect of weight change over a certain life period on LTL. To our knowledge, only two observational studies (28, 29) directly addressed the relation between weight change and LTL, but these studies were limited to samples that were obtained from women and were not representative of the entire nation.

This study aimed to investigate the relation between weight change across a 10-year period of adulthood **(**from a mean age of 41 years to 51 years) and LTL in a large sample that was nationally representative of the U.S. population. The patterns of weight change between two-time points were measured by BMI or absolute weight change. In addition, the analyses were stratified by sex and race/ethnicity to identify different effects within the corresponding subpopulation.

## Materials and Methods

### Study Population

We utilized the data from the National Health and Nutrition Examination Surveys (NHANES) 1999-2000 and 2001-2002 cycles. NHANES is a cross-sectional study designed to evaluate the health and nutritional status of U.S. adults and children and determine the prevalence of major diseases and risk factors. Data were collected by interviews, physical examination and laboratory testing, conducted by the U.S. National Center for Health Statistics (NCHS), a part of the Centers for Disease Control and Prevention (CDC). The detailed study design and data collection of NHANES are available at the online official website (30).

A total of 21,004 subjects from NHANES 1999-2002 cycle were enrolled in the present study. We incorporated 6,004 participants aged 36-75 years and sequentially excluded pregnant women (*n*=56), self-reported cancer patients (*n*=540), participants with energy intake less than 500 kcal or larger than 5,000 kcal (*n*=127), underweight participants (*n*=125) (BMI<18.5 kg/m^2^ at baseline or 10 years ago), individuals with missing data for BMI at baseline or self-reported weight at 10 years prior to the survey (*n*=599), or LTL (*n*=671). Finally, a total of 3,886 subjects were eligible for further analyses.

### Assessment of Weight Change

The baseline height and weight of the subjects were measured during physical examination. Respondents were asked to recall their weight at 10 years before the survey. BMIs at both time points were calculated as the corresponding weight (kg) divided by the square of baseline height (m^2^). BMI was further categorized into normal weight (<25.0), overweight (25.0-29.9), and obesity (≥30.0) (31). Five BMI change patterns were determined by BMIs at 10 years prior to the survey (BMI_10prior_) and at baseline (BMI_baseline_) (**Figure S1**):

- Stable normal weight (BMI<25.0 at both times);
- Normal weight to overweight or stable overweight (BMI_10prior_<30.0 to BMI_baseline_ from 25.0-29.9);
- Weight loss (BMI_10prior_≥30.0 to BMI_baseline_<30.0 or BMI_10prior_ in 25.0-29.9 to BMI_baseline_ <25.0);
- Non-obesity to obesity (BMI_10prior_<30.0 to BMI_baseline_≥30.0);
- Stable obesity (BMI≥30.0 at both time points).

We also generated new weight change patterns by classifying absolute weight change into five groups (27, 32): < -2.5 kg (weight loss), -2.5-2.5 kg (stable weight, reference group), 2.5-10.0 kg (mild weight gain), 10.0-20.0 kg (moderate weight gain) and ≥20.0 kg (severe weight gain).

### Telomere Length Measurement

All participants aged 20 years and older who had blood collected for DNA purification were eligible for baseline LTL measurements. LTL assay was performed and measured by quantitative polymerase chain reaction (qPCR) at the University of California, San Francisco. LTL was measured relative to standard reference DNA (T/S ratio), which was evaluated with samples from the human diploid fibroblast cell line IMR90. Each blood sample obtained from participants in NHANESs was assayed 3 times on 3 different days in duplicate wells that were blinded to the investigators. Sample plates were assayed in 3 groups, with no two plates grouped together more than once. Eight control DNA samples set in each assay plate were used to normalize between-run variability. If more than 4 control DNA values fell 2.5 standard deviations from the mean for all assay runs, they were excluded from further analysis (< 6% of runs). Any potential outliers in every sample were excluded. The mean and standard deviation of the T/S ratio were calculated for each sample. T/S ratio was furtherly converted to kilobase pairs (kbp) through the following formula: (3274 + 2413*(T/S))/1000, provided by NHANES analytic notes (33).

### Covariates

We categorize the collected data on three types of covariates in this study: demographic variables, lifestyle variables and medical comorbidities. The demographic variables included baseline age, sex, race/ethnicity, educational level, and family income-to-poverty ratio (PIR). Race/ethnicity was grouped into non-Hispanic white, non-Hispanic black, Mexican American and others, while educational level was classified as less than high school, high school or equivalent and college or above. Family PIR was calculated by dividing family income according to the poverty guidelines (34) and further divided into 3 categories (0-1.0, 1.1-3.0 and >3.0), representing low, medium, and high income, respectively. Lifestyle factors included energy intake, leisure-time physical activity, alcohol use (drinker, nondrinker) and smoking status (never, former, current smoker). Active physical activity was defined as at least 150 minutes of moderate activity, 75 minutes of vigorous activity or an equivalent combination of moderate and vigorous activity throughout the week. A drinker was defined as any participant who had at least 12 drinks of any type of alcoholic beverage in any one year. The medical comorbidities included self-reported health (excellent, good, poor), family history of diabetes (yes, no), family history of angina (yes, no), cardiovascular disease (yes, no), self-reported diabetes (yes, no) and self-reported hypertension (yes, no). If a participant was previously told that he/she had congestive heart failure, coronary heart disease, angina/angina pectoris, heart attack, or stroke, he/she was considered to have cardiovascular disease.

### Statistical Analyses

All the statistical analyses in this study took the complex survey design and sampling weights into consideration to form estimates that were representative of the U.S. civilian noninstitutionalized population. Data for population characteristics are presented as the mean and standard error (SE) for numerical variables, while the frequency (*n*) and proportion (%) for categorical variables are presented. Rao-Scott *χ* ^2^ test or weight-adjusted analysis of variance was employed to compare categorical or numerical baseline characteristics by weight change patterns, respectively. Missing values of the covariates were imputed using multivariate imputation by chained equations (MICE) to maintain statistical power. The number of imputations was set to 5, and the results of analyses on five imputed datasets were further combined. We calculated the Pearson correlation coefficient between BMIs at two-time points.

We first examined the associations between BMI categories at each time point and LTL. The normal BMI group was selected as the reference level. Afterward, analyses were mainly focused on the relation among five weight change patterns and LTL, in which maintaining a normal BMI pattern was regarded as the reference level. Survey-weighted linear regression was employed to infer the effects (coefficients) and 95% confidence interval for LTL in relation to BMI categories at two-time points and weight change patterns. Three models were built progressively to adjust for the possible confounding effects of different combinations of covariates. **Model 1** included the following covariates: baseline age, sex and race/ethnicity (non-Hispanic white, non-Hispanic black, Mexican American, and others). **Model 2** further included educational level (less than high school, high school or equivalent and college or above), family PIR (0-1.0, 1.1-3.0 and >3.0), physical activity (active, inactive), energy intake, alcohol use (yes, no) and smoking status (never, former, current and smoker). **Model 3** included self-reported health (excellent, good, poor), family history of diabetes (yes, no), family history of angina (yes, no), cardiovascular disease (yes, no), diabetes (yes, no) and hypertension (yes, no), in addition to the covariates in **Model 2**. We also evaluated the effect of absolute weight change on LTL during the time interval. In this analysis, baseline height and weight at 10 years before the survey were added as possible confounders to the three models. Furthermore, we investigated the associations between weight changes and LTL stratified by sex and race/ethnicity. A sensitivity analysis was performed to test the robustness of the results by removing subjects with missing values instead of performing imputations.

All analyses were performed in R (version 3.6.3) with packages *survey*, *mice* and *mitools*. All hypothesis tests were two-sided, and *P <*0.05 was considered statistically significant.

## Results

**Figure 1:**
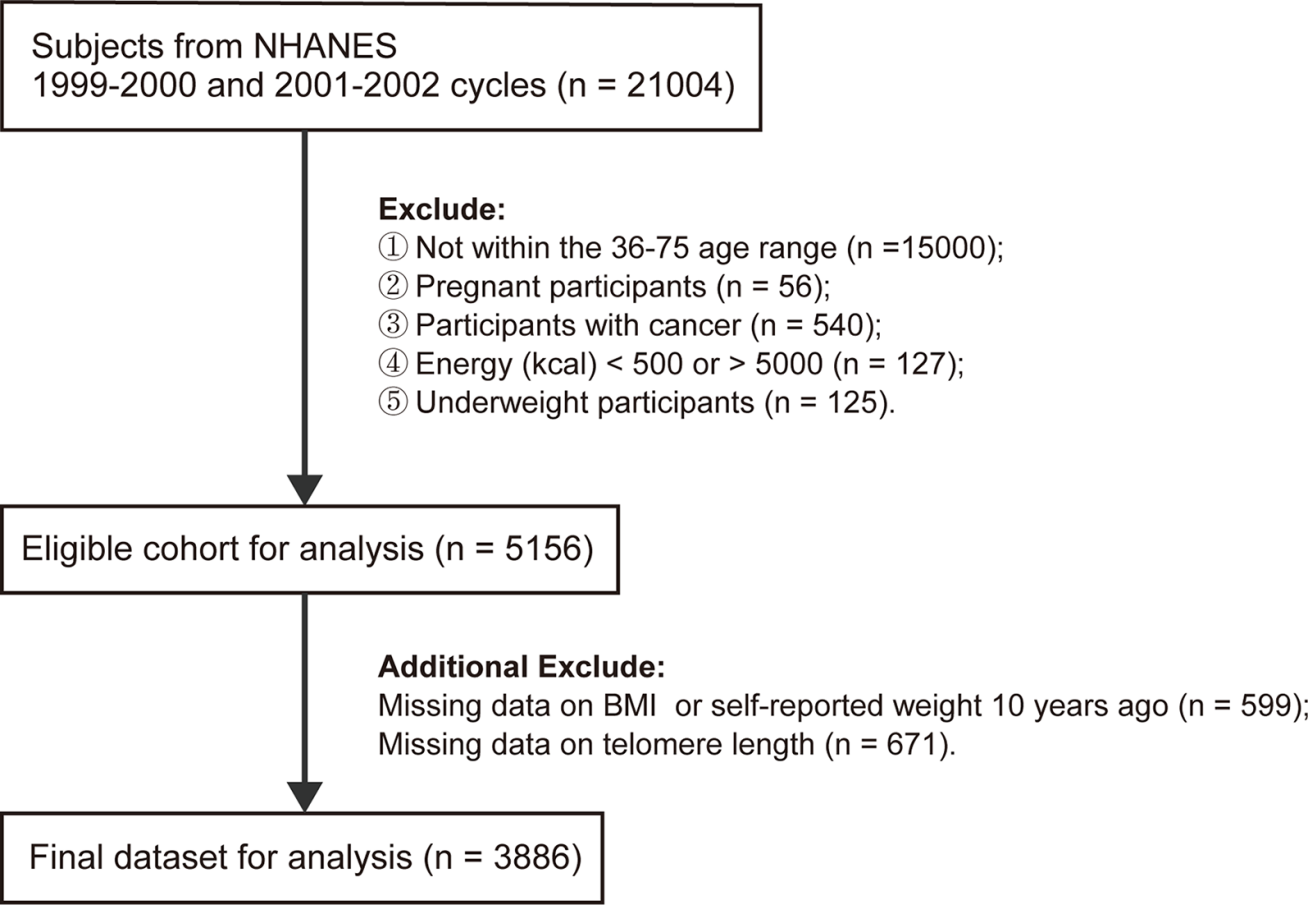
Flowchart of eligible subjects included in this study.

In total, 3,386 individuals were finally included in the subsequent analyses. A detailed flowchart is provided in **Figure 1**. The rates of missing covariates were less than 5.0%, except for PIR, which was 9.0%. The general characteristics of the total population are summarized in **Table 1**. The mean age (interquartile ranges) of individuals at the two-time points (i.e., baseline and 10 years before baseline) were 51 (42–59) and 41 (32–49) years, respectively. The average BMI increased from 26.3 to 28.8 during the 10 years before baseline. A high correlation (*r* = 0.68) was observed between BMI at the two-time points. Most people (46.1%) had normal BMIs in their early 40s and only 17.4% of the population was people with obesity. However, 10 years later, an increasing number of people were classified as overweight (37.2%) or obese (34.4%) (**Table S1**). During the 10-year period before the survey, 25.0% of the participants remained in the stable normal weight group, while 34.4% developed or maintained obesity. The proportions or means of each covariate were significantly different among the five weight change patterns. People who maintained obesity during the 10 years had the shortest LTL (5.67 kbp). LTL decreased steadily with age at a mean rate of 0.015 kbp (95% CI: 0.013-0.017), 0.016 kbp (95% CI: 0.013-0.018) and 0.014 kbp (95% CI: 0.011-0.018) per year for the general population, men and women, respectively (**Figure S2**).

**Table 1:** Baseline characteristics of study participants in the NHANES 1999-2002 cycle.

Characteristics	Total	Weight change patterns from 10 years ago to baseline					*P* value
		Stable normal weight	Normal weight to overweight or stay overweight	Weight losing	Non-obesity to obesity	Stable obesity	
Participants	3886 (100%)	830 (25.0%)	1331 (34.6%)	294 (6.0%)	798 (19.8%)	633 (14.6%)	
Sex							
Male	2053 (51.3%)	397 (43.1%)	811 (60.6%)	184 (62.4%)	354 (43.7%)	307 (49.2%)	<0.001
Female	1833 (48.7%)	433 (56.9%)	520 (39.4%)	110 (37.6%)	444 (56.3%)	326 (50.8%)	
Age (years)	50.9 (0.24)	49.3 (0.4)	50.9 (0.3)	53.8 (0.8)	51.0 (0.5)	52.5 (0.5)	<0.001
Race/ethnicity							
Non-Hispanic White	1873 (74.6%)	478 (79.1%)	646 (73.3%)	109 (72.0%)	350 (71.2%)	290 (75.6%)	<0.001
Non-Hispanic black	717 (9.1%)	122 (6.8%)	214 (7.9%)	61 (10.5%)	177 (11.7%)	143 (11.9%)	
Mexican American	977 (5.6%)	155 (3.8%)	345 (5.7%)	103 (7.0%)	206 (6.8%)	168 (6.2%)	
Others	319 (10.7%)	75 (10.2%)	126 (13.1%)	21 (10.4%)	65 (10.3%)	32 (6.3%)	
Education							
Less than high school	1342 (20.2%)	213 (13.4%)	467 (21.0%)	136 (30.0%)	282 (24.1%)	244 (21.4%)	<0.001
High school or equivalent	853 (25.2%)	168 (22.5%)	282 (25.5%)	62 (23.9%)	195 (28.3%)	146 (27.7%)	
College or above	1690 (54.7%)	449 (64.2%)	581 (54.5%)	96 (48.2%)	321 (47.7%)	243 (50.9%)	
PIR							
0-1.0	535 (10.3%)	104 (9.3%)	172 (8.9%)	53 (12.2%)	115 (12.2%)	91 (12.5%)	<0.001
1.1-3.0	1373 (30.8%)	240 (24.8%)	458 (29.4%)	123 (42.6%)	291 (32.0%)	261 (38.3%)	
>3.0	1629 (58.9%)	403 (66.0%)	600 (61.7%)	84 (45.2%)	324 (55.9%)	218 (49.2%)	
Activity							
Physically inactive	2522 (58.5%)	479 (50.0%)	816 (54.6%)	209 (65.4%)	551 (65.6%)	467 (69.7%)	<0.001
Physically active	1362 (41.5%)	351 (50.0%)	514 (45.4%)	85 (34.6%)	247 (34.4%)	165 (30.3%)	
Energy (kCal)	2145 (20)	2095 (37)	2234 (37)	2109 (84)	2051 (35)	2163 (48)	0.014
Alcohol Use							
Yes	2615 (74.0%)	589 (79.3%)	952 (78.0%)	206 (78.5%)	497 (66.3%)	371 (64.3%)	<0.001
No	1118 (26.0%)	205 (20.7%)	331 (22.0%)	74 (21.5%)	268 (33.7%)	240 (35.7%)	
Smoke							
No	1816 (46.4%)	374 (45.8%)	615 (45.3%)	119 (36.1%)	381 (47.9%)	327 (52.2%)	<0.001
Ever	1185 (30.2%)	207 (26.3%)	443 (33.2%)	76 (25.1%)	256 (30.4%)	203 (31.3%)	
Never	879 (23.4%)	249 (27.8%)	269 (21.5%)	99 (38.8%)	160 (21.7%)	102 (16.5%)	
Self-reported health							
Excellent	1754 (54.8%)	491 (68.9%)	676 (60.0%)	102 (47.9%)	292 (42.0%)	193 (38.5%)	<0.001
Good	1210 (28.7%)	203 (20.8%)	409 (28.1%)	87 (31.3%)	296 (35.8%)	215 (33.1%)	
Poor	920 (16.5%)	136 (10.3%)	246 (11.9%)	105 (20.8%)	209 (22.2%)	224 (28.4%)	
Family History Diabetes							
Yes	1940 (50.1%)	348 (40.6%)	635 (47.4%)	159 (52.9%)	432 (55.2%)	366 (64.5%)	<0.001
No	1893 (49.9%)	470 (59.4%)	678 (52.6%)	130 (47.1%)	355 (44.8%)	260 (35.5%)	
Family History Angina							
Yes	471 (14.7%)	88 (12.4%)	152 (14.4%)	34 (12.0%)	98 (14.7%)	99 (20.2%)	0.038
No	3324 (85.3%)	724 (87.6%)	1148 (85.6%)	254 (88.0%)	680 (85.3%)	518 (79.8%)	
CVD							
Yes	409 (8.6%)	46 (4.0%)	118 (8.1%)	48 (13.6%)	92 (10.1%)	105 (13.8%)	<0.001
No	3458 (91.4%)	783 (96.0%)	1206 (91.9%)	244 (86.4%)	700 (89.9%)	525 (86.2%)	
Diabetes							
Yes	451 (8.1%)	29 (2.4%)	107 (5.1%)	85 (20.4%)	81 (8.2%)	149 (20.2%)	<0.001
No	3361 (91.9%)	793 (97.6%)	1201 (94.9%)	200 (79.6%)	698 (91.8%)	469 (79.8%)	
Hypertension							
Yes	1317 (29.2%)	148 (13.5%)	378 (24.9%)	122 (34.4%)	335 (40.2%)	334 (49.6%)	<0.001
No	2549 (70.8%)	678 (86.5%)	944 (75.1%)	171 (65.6%)	461 (59.8%)	295 (50.4%)	
Telomere length (kbp)	5.75 (0.04)	5.85 (0.04)	5.74 (0.04)	5.70 (0.05)	5.72 (0.05)	5.67 (0.04)	<0.001
BMI at baseline	28.8 (0.20)	22.5 (0.07)	27.4 (0.04)	25.6 (0.19)	33.5 (0.11)	37.9 (0.32)	<0.001
BMI at 10 years ago	26.3 (0.14)	21.8 (0.08)	25.1 (0.08)	29.5 (0.33)	26.7 (0.10)	35.2 (0.32)	<0.001

PIR, Poverty Income Ratio; CVD, Cardiovascular Disease.

Data for population characteristics are presented as the mean and standard error (SE) for numerical variables and the frequency (n) and proportion (%) for categorical variables. The P value was calculated with the Rao-Scott χ2 test or by weight-adjusted analysis of variance.

**Figure S3** shows the linear relation between LTL and weight status at each time point. Compared with LTL of the participants with normal weights at 10 years before baseline, on average, the LTL of people with overweight and obesity, respectively were 0.073 kbp (95% CI: 0.039-0.107, *P*=3.11E-05) and 0.110 kbp (95% CI: 0.063-0.156, *P*=3.40E-06) shorter in the fully adjusted Model (e.g., **Model 3**), which was consistent with the results in **Models 1**, **2**. A similar trend in the association was observed between LTL and baseline BMI categories.

**Figure 2:**
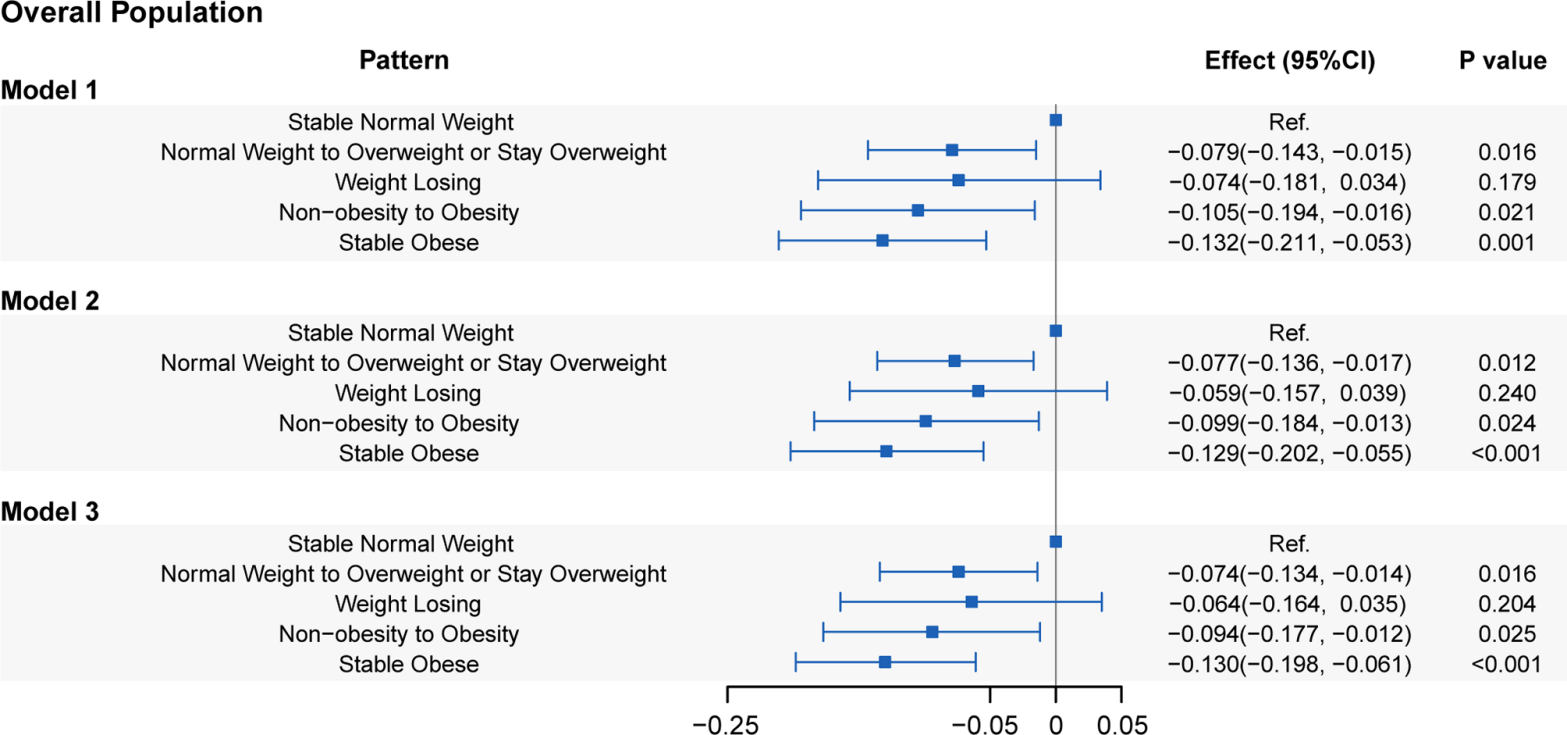
Associations of leucocyte telomere length with five weight change patterns. **Model 1**: adjusted for age, sex, and race/ethnicity. **Model 2**: adjusted for covariates in **Model 1** plus educational level, family PIR, physical activity, energy intake, alcohol use, and smoking status. **Model 3**: adjusted for covariates in **Model 2** plus self-reported health, family history of diabetes, family history of angina, cardiovascular disease, diabetes, and hypertension.

The relation between weight change patterns during the 10-year period before baseline and LTL in the three models is presented in **Figure 2**. **Model 3** showed that people with stable obesity had a 0.130 kbp (95% CI: 0.061-0.198, *P*=1.97E-04) shorter LTL than those with stable normal weight (reference group). Weight gain from non-obesity to obesity shortened the LTL by 0.094 kbp (95% CI: 0.012-0.177, *P*=0.026), while moving from normal weight to overweight or maintaining overweight shortened the LTL by 0.074 kbp (95% CI: 0.014-0.134, *P*=0.016). In addition, the LTL of weight loss (i.e., from obesity to non-obesity or from overweight to normal weight) was not significantly different from that of stable normal weight (coefficient: -0.064; 95% CI: -0.164–0.035; *P*=0.204). **Figure S4** shows that stable obesity during the period was related to a shorter LTL in both male and female populations. All of the other weight change patterns shortened the LTL compared with stable normal weight in males but did not yield a statistically significant effect on LTL in females. Compared with those with a stable normal weight, non-Hispanic white individuals, with weight change patterns except for weight loss, had significantly shorter LTLs, whereas only non-Hispanic black individuals with stable obesity had a shortened LTL, by 0.150 kbp (95% CI: 0.032-0.267, *P*=0.012) (**Figure S5**). Moreover, among Mexican Americans, there were no differences in LTLs observed among the five weight change patterns.

**Figure 3:**
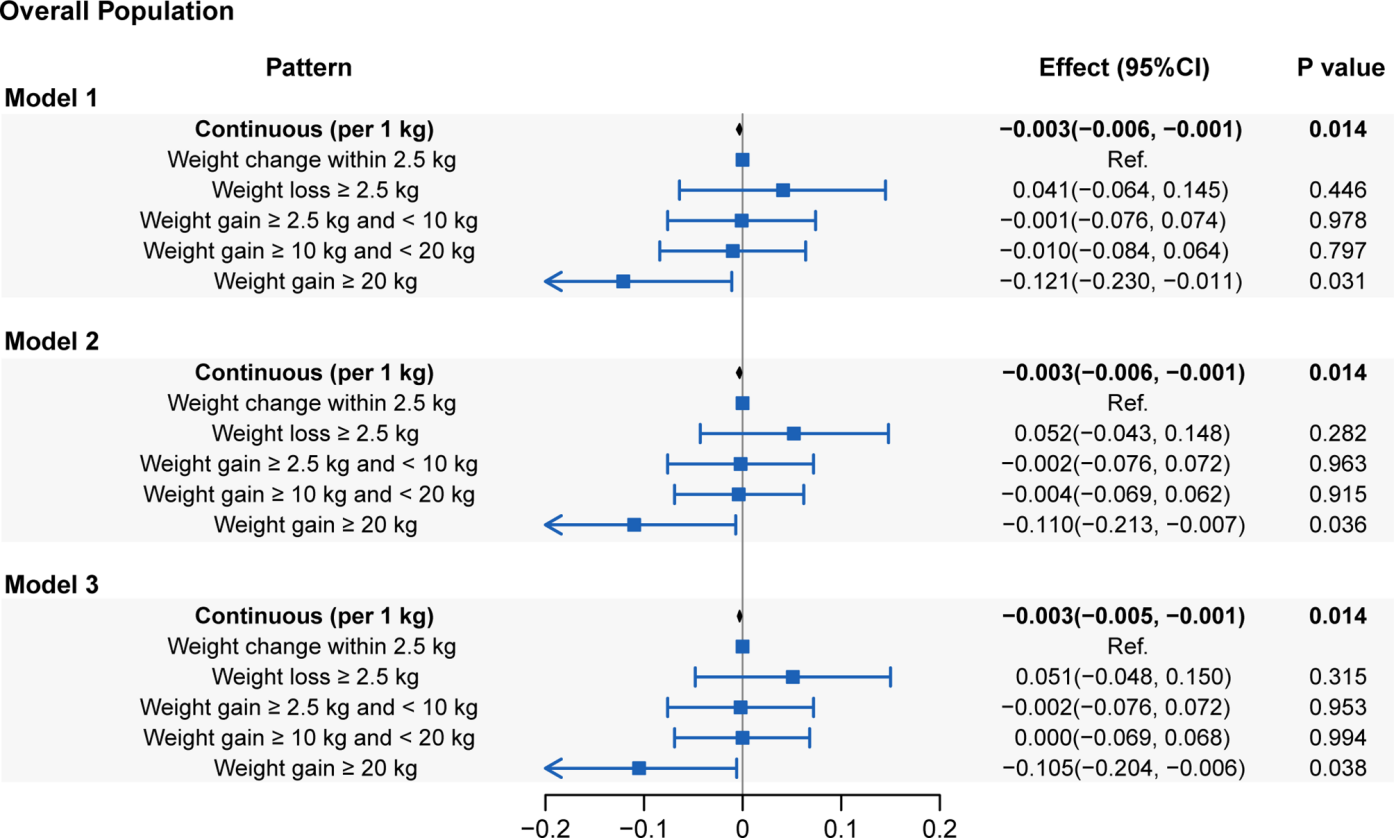
Associations of leucocyte telomere length with absolute weight change (presenting as continuous and categorical forms). **Model 1**: adjusted for age, sex and race/ethnicity. **Model 2**: adjusted for covariates in **Model 1** plus educational level, family PIR, physical activity, energy intake, alcohol use and smoking status. **Model 3**: adjusted for covariates in **Model 2** plus self-reported health, family history of diabetes, family history of angina, cardiovascular disease, diabetes and hypertension.

In the evaluation of the associations between the absolute weight change at the two-time points and LTL, a linear attenuation of the LTL with weight change was observed (coefficient: -0.003 kbp/kg; 95% CI: -0.006–0.001; *P*=0.014). When further classified, weight gain ≥ 20.0 kg across the 10-year period shortened the LTL by 0.105 kbp (95% CI: 0.006-0.204, *P*=0.038) in **Model 3** compared with a weight change within 2.5 kg (**Figure 3**). Additionally, there was no significant difference in the LTL of weight gain of either 10.0-20.0 kg or 2.5-10 kg compared to the LTL of weight change within 2.5 kg (*P*=0.953 and *P*=0.994, respectively). Weight loss > 2.5 kg presented a sign of having a longer LTL (0.051 kbp, 95% CI: -0.048-0.150) than weight change within 2.5 kg, but the difference was not statistically significant (*P*=0.351).

The results of and were generally consistent with those of in each analysis. Further sensitivity analysis using complete cases attenuated the associations, but the most significant results remained.

## Discussion

In this large cross-sectional study of nationally representative U.S. adults, we showed the elevated long-term impact of weight status on LTL in adulthood, with the ages ranging from 36 to 75 years. People with stable normal weight during the 10 years before baseline (mean age, 51 years) had the longest LTL among the five weight change patterns, while the LTL of people with stable obesity was shortest. Further stratified analysis indicated that all associations had sex differences, but people who maintain obesity show a consistent effect. Variations across races/ethnicity were also observed. When using the absolute weight change, we found that the LTL linearly decreased with weight gain. The findings suggested the importance of keeping a normal weight to maintain a long LTL, a biomarker of aging-related disorders.

Our study suggested that the difference in LTL between individuals with a stable normal weight and those with stable obesity corresponds to approximately 8.7 years of aging, while weight gain from non-obesity to obesity corresponds to approximately 6.3 years of aging. The distinction in LTL between weight loss and having a stable normal weight was not statistically significant, suggesting that weight loss could also be beneficial for telomere protection. The result of modeling with absolute weight change indicated that an additional 1 kg of weight seemed to accelerate as much as 0.2 years of aging. Stratified analysis showed sex and race/ethnicity variations in the strength of associations. Specifically, males with overweight or obesity of the two-time points had significantly shorter LTLs than those with stable normal weight, whereas only maintaining obesity shortened the LTL in females. Among non-Hispanic whites, the influence of weight change on LTL was more remarkable than that among the other races/ethnicities. The generally identical results derived from the three models with different covariate adjustments demonstrate the robustness of our conclusions. Regarding the possible limitation of reduced sample size, the sensitivity analysis with complete cases attenuated the associations, but the most significant associations remained.

The current mainstream mechanism explaining chronic inflammation-induced telomere dysfunction is oxidative stress, an imbalance between the production of reactive oxygen species(ROS) and cellular antioxidant defenses (35). Increasing obesity may result in oxidative stress (36), and oxidative damage anywhere in the telomere can cause stochastic and irregular telomere shortening events in human fibroblasts (37). Another study demonstrated that 7,8-dihydro-8-oxoguanine (8-oxoG) is the most predominant lesion caused by oxidative stress, while the chronic formation of 8-OxoG is found to triggers telomere losses (38). A recent review of evidence from humans, mouse models and cell culture studies showed that oxidative stress is correlated with accelerated telomere shortening and dysfunction (39). The observed correlation between obesity and LTL might also be explained by the fat mass and obesity associated (*FTO*) gene-involved pathways, as is shown in the review (40). Genome-wide association studies have identified *FTO* as an obesity-associated gene (41, 42). *FTO* rs8050136 was found to correlate with the expression of retinoblastoma-like 2 protein (Rbl2) gene (43), which inhibits Dnmt3a,3b expression, thus influencing telomere regulation process (44).

There are a few studies investigating the relation between weight changes and LTL. Kim et al. (28) explored the association between weight gain and TL (relative T/S ratio) among 608 women aged ≥ 40 years enrolled in the NIEHS Sister Study, where weight gain was defined as the difference between current and past weight (i.e., self-reported average weight at ages 30 to 39) and further classified into five patterns. They showed that overweight (BMI ≥ 25 kg/m^2^) or obesity (BMI ≥30 kg/m^2^) at both time points had the smallest T/S ratio and a maintained normal BMI had the longest TL, which was consistent with our results. However, this study included only women and an insufficient number of samples (only 3 available participants) to address the influence of weight loss on TL. Cui et al. (29) defined weight change patterns based on recalled weight and measured weight at enrollment, reporting its inverse association with TL in a study of 1,295 women aged 55 to 70 years. Specifically, they classified BMI changes into five groups and showed that women who maintained a normal weight or reduced their weight to normal since the age of 50 had a longer TL than those with stable obesity or those who became obese. Compared with their study, our study included a nationally representative sample comprising both males and females with a wide age range and presents sex and race/ethnicity differences in the associations. In addition, we present the stability of our results by considering the different combinations of covariates and sensitivity analysis.

Several limitations in this study should be addressed. Firstly, we employed BMI as the only measurement of adiposity because the data collected from NHANES lacked other adiposity-related markers, such as body fat and waist circumference, at 10 years before baseline; therefore, our conclusions, derived from BMI, are incomplete. Secondly, data on many covariates (e.g., physical activity) were collected only at baseline, thus ignoring changes over the 10-year interval, which makes it difficult to control for time-varying confounders. Moreover, it is difficult to make causal inferences in this cross-sectional study and self-reported weight data we used in this study inevitably carries the risk of recall bias.

The development of telomere biology has opened up a new path toward understanding the mechanisms related to aging, obesity and oxidative stress at the molecular level. Our study explored the association between weight change patterns and LTL based on a large sample size, which further supports the theory that gaining weight promotes aging. LTL can be used as a biomarker for obesity treatment, warning people to intervene as soon as possible to reduce the risk of obesity-related diseases and maintain a normal weight.

## Data Availability Statement

Publicly available datasets were analyzed in this study. This data can be found here: https://wwwn.cdc.gov/nchs/nhanes/Default.aspx.

## Ethics Statement

The studies involving human participants were reviewed and approved by the NCHS Research Ethics Review Board (#98-12). The patients/participants provided their written informed consent to participate in this study.

## Author Contributions

YZ and WD conceptualized the study. YZ, ZX, YY, SC, and WD analyzed and interpreted the data, and drafted the article. SL provided valuable suggestions for study design and data analysis. WD supervised the study. All authors contributed to the article and approved the submitted version.

## Funding

This research was supported by grants from National Natural Science Foundation of China (Grant No. 81903409), General Project of Science and Technology Development Fund of Nanjing Medical University (NMUB2018020), Development Program of Medical Big Data Analysis Software of Nanjing Medical University (Grant No. 2019KF0106).

## Conflict of Interest

The authors declare that the research was conducted in the absence of any commercial or financial relationships that could be construed as a potential conflict of interest.

## Publisher’s Note

All claims expressed in this article are solely those of the authors and do not necessarily represent those of their affiliated organizations, or those of the publisher, the editors and the reviewers. Any product that may be evaluated in this article, or claim that may be made by its manufacturer, is not guaranteed or endorsed by the publisher.
