# Supplementary material for: Association Between Weight Change and Leukocyte Telomere Length in U.S. Adults
Source: Front Endocrinol (Lausanne). 2021 Jul 28;12:650988. doi: 10.3389/fendo.2021.650988 (PMC8355991; doi:10.3389/fendo.2021.650988)
Supplement: Supplementary file 1 [file DataSheet_1.docx]

**Supplementary Material**

**Table S1:** Baseline characteristics of the study participants in the NHANES 1999-2002 cycle according to BMI categories at two time points (10 years before survey and baseline).

Data for population characteristics are presented as the mean and standard error (SE) for numerical variables and the frequency (n) and proportion (%) for categorical variables. The *P* value was calculated with Rao-Scott χ2 test or by weight-adjusted analysis of variance.

**Figure S1:** Five BMI change patterns defined by BMIs at 10 years prior to the survey and at baseline.

**Figure S2:** The linear relationships between telomere length (kbp) and baseline age (years) for the general population, men and women.

**Figure S3:** The associations between BMI categories and telomere length at two time points (10 years before survey and baseline)

**Figure S4:** Stratification analysis of the associations of leucocyte telomere length with five weight change patterns by sex.

Model 1: adjusted for age, race/ethnicity. Model 2: adjusted for covariates in Model 1 plus educational level, family PIR, physical activity, energy intake, alcohol use, and smoking status. Model 3: adjusted for covariates in Model 2 plus self-reported health, family history of diabetes, family history of angina, cardiovascular disease, diabetes, and hypertension.

**Figure S5:** Stratification analysis of the associations of leucocyte telomere length with weight change patterns by race/ethnicity.

Model 1: adjusted for age, sex. Model 2: adjusted for covariates in Model 1 coupled with educational level, family PIR, physical activity, energy intake, alcohol use, and smoking status. Model 3: adjusted for covariates in Model 2 plus self-reported health, family history of diabetes, family history of angina, cardiovascular disease, diabetes, and hypertension.

**Table S1:** Baseline characteristics of the study participants in the NHANES 1999-2002 cycle according to BMI categories at two time points (10 years before survey and baseline). Data for population characteristics are presented as the mean and standard error (SE) for numerical variables and the frequency (*n*) and proportion (%) for categorical variables. The *P* value was calculated with the Rao-Scott χ2 test or by weight-adjusted analysis of variance.

| Variables | BMI category at 10 years ago | | | | | *P* value | BMI category at baseline | | | *P* value |
| --- | --- | --- | --- | --- | --- | --- | --- | --- | --- | --- |
|  | Normal weight  (<25.0) | | | Overweight  (25.0~29.9) | Obesity  (≥30.0) |  | Normal weight  (<25.0) | Overweight  (25.0~29.9) | Obesity  (≥30.0) |  |
| Sex |  | | |  |  |  |  |  |  |  |
| Male | 698(41.4%) | | | 948(63.7%) | 407(51.5%) | <0.001 | 492(45.4%) | 900(60.7%) | 661(46.0%) | <0.001 |
| Female | 882(58.6%) | | | 566(36.3%) | 385(48.5%) |  | 490(54.6%) | 573(39.3%) | 770(54.0%) |  |
| Age (years) | 48.9(0.3) | | | 52.7(0.3) | 52.7(0.5) | <0.001 | 49.9(0.4) | 51.1(0.3) | 51.6(0.4) | <0.001 |
| Race |  | | |  |  |  |  |  |  |  |
| Non-Hispanic White | 827(75.0%) | | | 706(74.0%) | 340(74.9%) | <0.001 | 542(78.2%) | 691(73.3%) | 640(73.0%) | <0.001 |
| Non-Hispanic black | 283(8.5%) | | | 256(8.6%) | 178(11.9%) |  | 152(7.2%) | 245(8.1%) | 320(11.8%) |  |
| Mexican American | 318(4.4%) | | | 426(6.6%) | 233(6.7%) |  | 200(4.0%) | 403(6.0%) | 374(6.5%) |  |
| Others | 152(12.1%) | | | 126(10.9%) | 41(6.5%) |  | 88(10.6%) | 134(12.7%) | 97(8.6%) |  |
| Education |  | | |  |  |  |  |  |  |  |
| Less than high school | 466(23.4%) | | | 546(22.0%) | 330(17.5%) | 0.004 | 271(14.5%) | 545(22.0%) | 526(22.9%) | <0.001 |
| High school or equivalent | 343(24.3%) | | | 336(25.7%) | 174(26.4%) |  | 206(23.3%) | 306(24.0%) | 341(28.0%) |  |
| College or above | 771(58.2%) | | | 631(52.3%) | 288(50.2%) |  | 505(62.2%) | 621(54.1%) | 564(49.1%) |  |
| PIR |  | | |  |  |  |  |  |  |  |
| 0-1.0 | 223(10.8%) | | | 190(8.9%) | 122(12.3%) | <0.001 | 128(9.7%) | 201(9.0%) | 206(12.3%) | 0.001 |
| 1.1~3.0 | 510(28.4%) | | | 523(29.6%) | 331(39.9%) |  | 303(26.5%) | 518(30.6%) | 552(34.6%) |  |
| >3.0 | 712(60.8%) | | | 660(61.6%) | 257(47.8%) |  | 451(63.8%) | 636(60.4%) | 542(53.1%) |  |
| Activity |  | | |  |  |  |  |  |  |  |
| Physically inactive | 984(56.0%) | | | 957(56.9%) | 581(68.4%) | <0.001 | 589(52.4%) | 915(55.0%) | 1018(67.4%) | <0.001 |
| Physically active | 596(44.0%) | | | 556(43.1%) | 210(31.6%) |  | 393(47.6%) | 557(45.0%) | 412(32.6%) |  |
| Energy (kCal) | 2090(26) | | | 2214(33) | 2146(47) | 0.008 | 2102(35) | 2221(36) | 2099(29) | 0.023 |
| Alcohol Use |  | | |  |  |  |  |  |  |  |
| Yes | 1100(76.5%) | | | 1035(74.5%) | 480(66.6%) | 0.005 | 699(79.2%) | 1048(78.0%) | 868(65.4%) | <0.001 |
| No | 419(23.5%) | | | 417(25.5%) | 282(33.4%) |  | 239(20.8%) | 371(22.0%) | 508(34.6%) |  |
| Smoke |  | | |  |  |  |  |  |  |  |
| No | 714(45.0%) | | | 713(47.1%) | 389(48.5%) | <0.001 | 437(45.1%) | 671(44.3%) | 708(49.7%) | <0.001 |
| Ever | 418(26.9%) | | | 519(34.1%) | 248(30.6%) |  | 241(25.9%) | 485(32.9%) | 459(30.8%) |  |
| Never | 445(28.1%) | | | 280(18.7%) | 154(20.9%) |  | 304(29.0%) | 313(22.8%) | 262(19.5%) |  |
| Self-reported health |  | | |  |  |  |  |  |  |  |
| Excellent | 855(62.8%) | | | 663(52.5%) | 236(38.6%) | <0.001 | 554(67.4%) | 715(58.4%) | 485(40.5%) | <0.001 |
| Good | 432(23.9%) | | | 510(32.3%) | 268(33.8%) |  | 243(21.3%) | 456(28.9%) | 511(34.6%) |  |
| Poor | 293(13.3%) | | | 340(15.2%) | 287(27.6%) |  | 185(11.3%) | 302(12.7%) | 433(24.8%) |  |
| Family History Diabetes | | |  |  |  |  |  |  |  |  |
| Yes | 722(45.0%) | | | 756(50.3%) | 462(62.9%) | <0.001 | 421(42.0%) | 721(47.9%) | 798(59.1%) | <0.001 |
| No | 836(55.0%) | | | 737(49.7%) | 320(37.1%) |  | 547(58.0%) | 731(52.1%) | 615(40.9%) |  |
| Family History Angina | |  | |  |  |  |  |  |  |  |
| Yes | 181(14.3%) | | | 174(13.1%) | 116(19.0%) | 0.089 | 106(12.3%) | 168(14.3%) | 197(17.1%) | 0.060 |
| No | 1362(85.7%) | | | 1306(86.9%) | 656(81.0%) |  | 856(87.7%) | 1270(85.7%) | 1198(82.9%) |  |
| CVD |  | | |  |  |  |  |  |  |  |
| Yes | 92(4.7%) | | | 187(11.1%) | 130(13.8%) | <0.001 | 72(5.3%) | 140(8.3%) | 197(11.7%) | <0.001 |
| No | 1485(95.3%) | | | 1315(88.9%) | 658(86.2%) |  | 908(94.7%) | 1325(91.7%) | 1225(88.3%) |  |
| Diabetes |  | | |  |  |  |  |  |  |  |
| Yes | 66(2.9%) | | | 178(8.4%) | 207(21.6%) | <0.001 | 64(3.9%) | 157(6.7%) | 230(13.3%) | <0.001 |
| No | 1497(97.1%) | | | 1299(91.6%) | 565(78.4%) |  | 905(96.1%) | 1289(93.3%) | 1167(86.7%) |  |
| Hypertension |  | | |  |  |  |  |  |  |  |
| Yes | 344(18.7%) | | | 561(33.3%) | 412(48.6%) | <0.001 | 200(15.3%) | 448(26.1%) | 669(44.2%) | <0.001 |
| No | 1229(81.3%) | | | 944(66.7%) | 376(51.4%) |  | 777(84.7%) | 1016(73.9%) | 756(55.8%) |  |
| Telomere length (kbp) | 5.83(0.04) | | | 5.69(0.04) | 5.67(0.04) | <0.001 | 5.84(0.04) | 5.73(0.04) | 5.70(0.04) | 0.004 |
| BMI at baseline | 25.1(0.1) | | | 29.9(0.1) | 36.3(0.4) | <0.001 | 22.5(0.1) | 25.6(0.1) | 30.3(0.2) | <0.001 |

PIR: Poverty Income Ratio; CVD: Cardiovascular Disea
