# Supplementary figures and images for: Association Between Weight Change and Leukocyte Telomere Length in U.S. Adults
Source: Front Endocrinol (Lausanne). 2021 Jul 28;12:650988. doi: 10.3389/fendo.2021.650988 (PMC8355991; doi:10.3389/fendo.2021.650988)

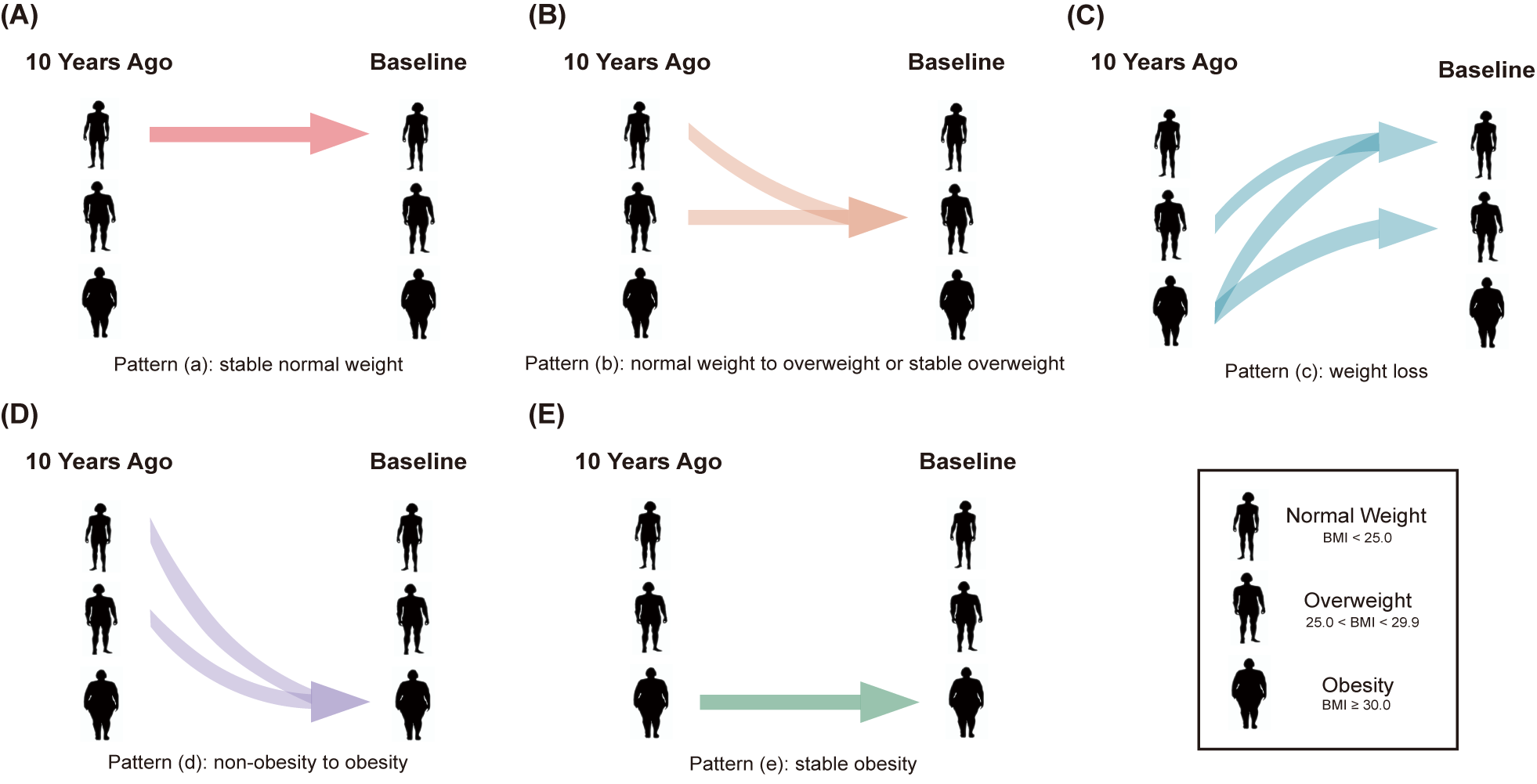

Supplement: Supplementary file 2 [file Image_1.tif]

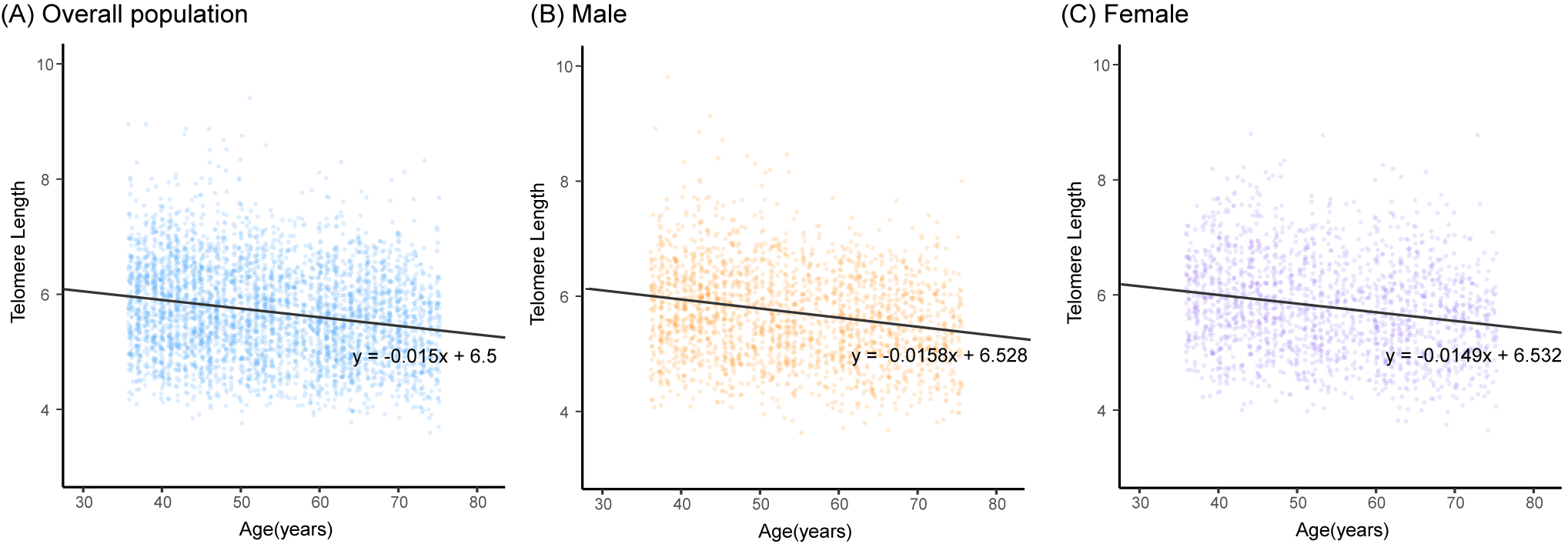

Supplement: Supplementary file 3 [file Image_2.tif]

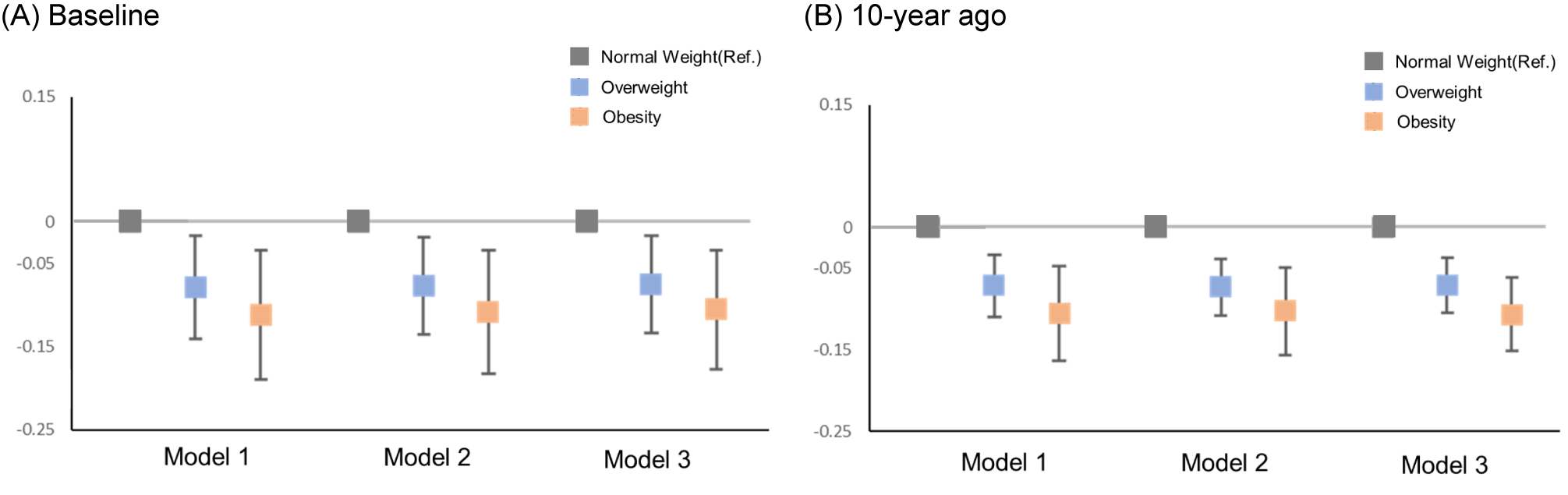

Supplement: Supplementary file 4 [file Image_3.tif]

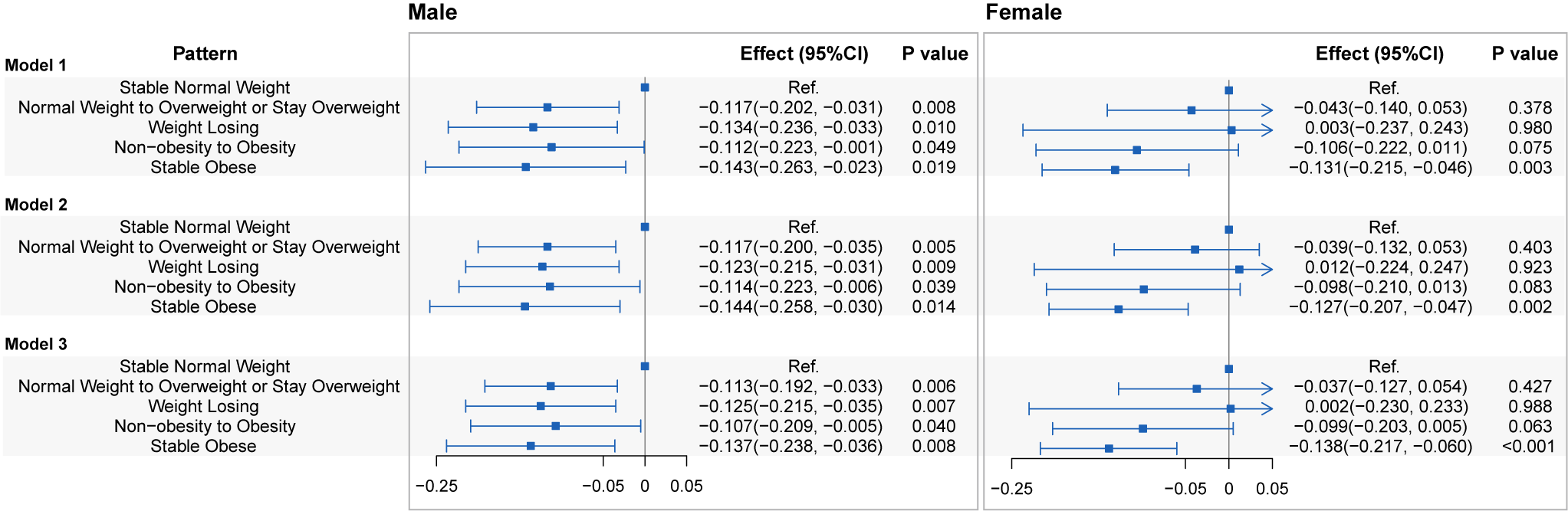

Supplement: Supplementary file 5 [file Image_4.tif]

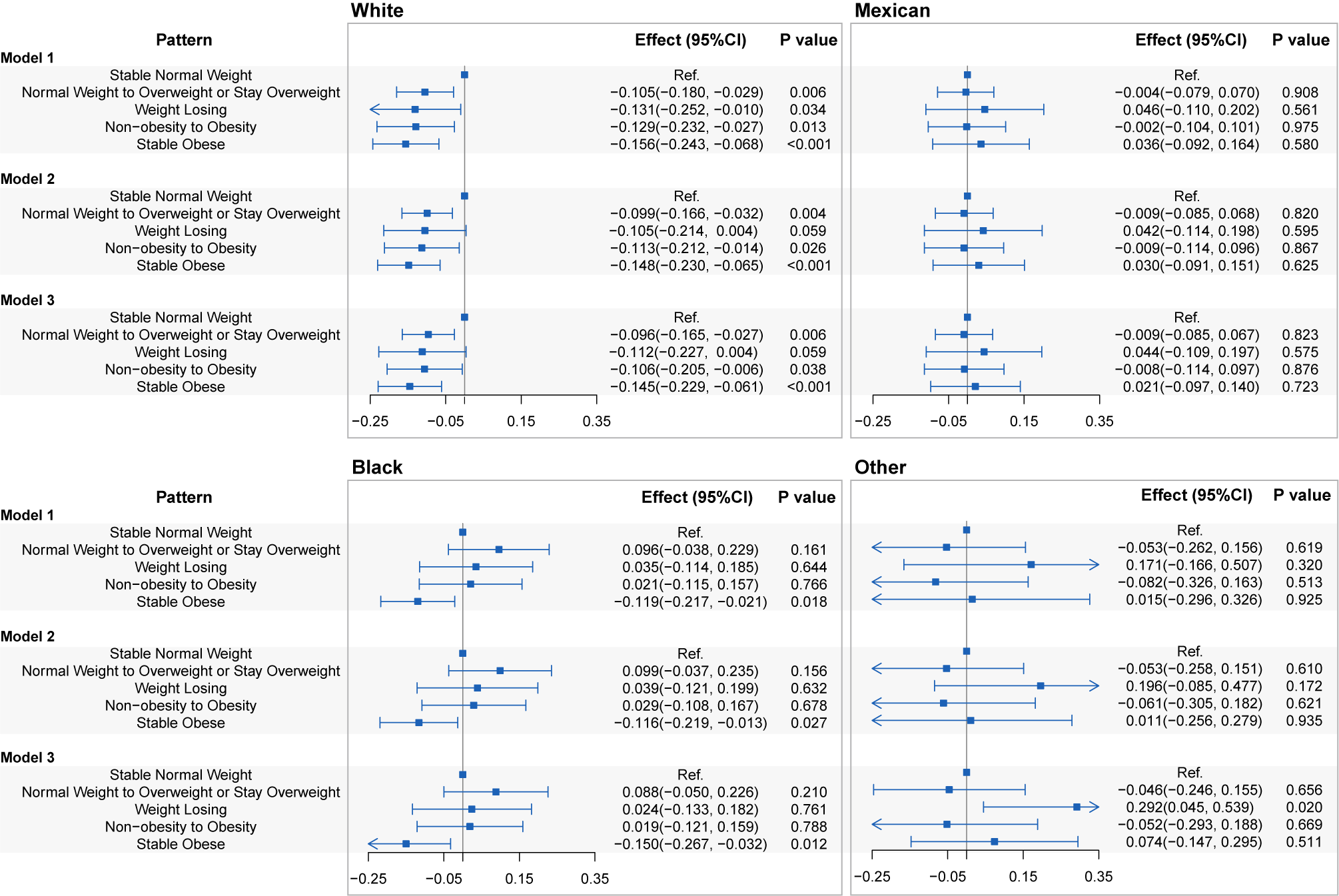

Supplement: Supplementary file 6 [file Image_5.tif]
